# Optimal Antiviral Switching to Minimize Resistance Risk in HIV Therapy

**DOI:** 10.1371/journal.pone.0027047

**Published:** 2011-11-03

**Authors:** Rutao Luo, Michael J. Piovoso, Javier Martinez-Picado, Ryan Zurakowski

**Affiliations:** 1 Department of Electrical and Computer Engineering, University of Delaware, Newark, Delaware, United States of America; 2 Department of Electrical Engineering, Penn State University Great Valley, Malvern, Pennsylvania, United States of America; 3 Institut de Recerca de la SIDA, IrsiCaixa, Badalona, Spain; 4 Institució Catalana de Recerca i Estudis Avançats (ICREA), Barcelona, Spain; 5 Delaware Biotechnology Institute, Newark, Delaware, United States of America; University of Illinois at Urbana-Champaign, United States of America

## Abstract

The development of resistant strains of HIV is the most significant barrier to effective long-term treatment of HIV infection. The most common causes of resistance development are patient noncompliance and pre-existence of resistant strains. In this paper, methods of antiviral regimen switching are developed that minimize the risk of pre-existing resistant virus emerging during therapy switches necessitated by virological failure. Two distinct cases are considered; a single previous virological failure and multiple virological failures. These methods use optimal control approaches on experimentally verified mathematical models of HIV strain competition and statistical models of resistance risk. It is shown that, theoretically, order-of-magnitude reduction in risk can be achieved, and multiple previous virological failures enable greater success of these methods in reducing the risk of subsequent treatment failures.

## Introduction

The development of multi-drug regimens for HIV therapy has resulted in HIV infection becoming a chronic, manageable disease in first world countries [1]. The necessity of a three-drug regimen, where each drug in the regimen targets separate viral epitopes, is due to the extremely high replication and mutation rates characteristic of HIV infection [2]. These make the evolution of viral strains resistant to a single drug inevitable. Three drugs, however, present a mutational barrier high enough to make such an evolutionary occurrence unlikely [3], [4]. These combinations contain three drugs from at least two separate classes of antivirals, including the nucleoside/nucleotide analog reverse-transcriptase inhibitors (nRTI), non-nucleoside reverse-transcriptase inhibitors (NNRTI), protease inhibitors (PI), and integrate inhibitors (II). While these three- drug regimens, known as highly active antiretroviral therapy, or HAART, are highly effective at suppressing the virus in the long term, some patients nevertheless experience viral load rebound, driven by the emergence of a viral mutant resistant to all three components of their HAART regimen.

### Mutation

Mutation events in HIV replication appear to be dominated by point-substitution events, which occur with very high frequency. This, coupled with the high turnover rate of HIV in uncontrolled infection, create a situation in which multi-drug resistant virus develops frequently. When a resistant mutant emerges, it becomes necessary to switch to a new three-drug regimen, whose components exhibit no cross-resistance with the failed three-drug regimen [5]. There are a limited number of independent drug combinations. A patient who has developed viral strains resistant to all such combinations is called Multi-Drug Resistant or MDR, and such patients are left with few viable treatment options. It is critical, therefore, to preserve the remaining pool of independent HAART regimens, especially for patients who have experienced virological failure on more than one previous regimen.

Attempts have been made to re-sensitize the virus to previously failed regimens through the use of treatment interruptions; the theory is that the wild-type virus, which enjoys a competitive advantage in the absence of therapy, would re-establish dominance and potentially drive the resistant virus extinct through competition [6]. Although these studies showed a brief return of susceptibility, the resistant strain quickly returned upon re-introduction of the drug regimen, and overall patient outcome was worse than a non-interrupted control group. More recent approaches have focused on changing the genetic makeup of the viral pool in MDR patients in preparation for 4-9 drug rescue regimens known as Mega-HAART or giga-HAART [7], [8], [9], [10], [11], [12]. These approaches showed mixed results, mostly with poor clinical outcomes. All of these previous approaches attempted to use treatment interruptions to manipulate the susceptibility of the virus to regimens consisting of drugs to which resistant virus had already emerged. None of these addressed the possibility of using interruptions to preserve the usefulness of a naive antiviral regimen. Also, the antiviral regimen introduced following the interruption was always novel, implying an attempt to manipulate susceptibility by genetic profile alone, as opposed to manipulating viral load and genetic profile.

Attempts have also been made to use previously failed drugs in novel combinations in order to preserve some usefulness from previously failed treatments in MDR patients. The problem with this, however, is that the existing mutations represent a lowering of the mutational barrier. The only way to overcome this in the long-term seems to be an increase in the number of drug components used, which may succeed at the goal of reducing viral load at the cost of raising the side effects of the HAART drugs to an unacceptable level. A notable exception to this was the recent Tetriz study [13]. In this study, a drug combination using components from previously failed regimens, including two drugs for which the resistance mutations were known to be antagonistic. Despite the use of only four previously failed components, this regimen succeeding in inducing complete viral suppression in a significant portion of the study group, strongly suggesting the usefulness of permuted regimens. The importance of preserving suppressive regimens has driven a number of clinical studies, including the SWATCH study [14], which showed reduced incidence of virological failure in patients undergoing a pre-emptive switching schedule based on mathematical models of risk similar to those described in the Analysis section.

### Competition and Selection

The development of drug resistance in HIV infection is driven by two phenomenon: mutation and selection. Mutation in HIV replication occurs at a well-characterized, relatively constant rate of approximately 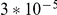 substitutions per base-pair per replication cycle [15]. Other mutation types, such as deletions, insertions, and rotations, happen with considerably lower frequency, and do not usually contribute to the development of resistance. Despite this relatively high rate of mutation, the population of virus in a treatment-naive patient contains only virus with very few genetic changes from the nominal, or “wild-type” HIV sequence. This is due to the influence of selection; the wild-type dominates in the absence of treatment because it is usually the fittest virus in that environment. Many mutations carry a fitness cost when compared to the wild-type sequence; viruses carrying these mutations do not replicate as efficiently as the wild-type virus. The various virus subtypes compete for target cells, so selective pressures tend to drive extinct virus variants that carry mutations.

When antiviral medication is used, the wild-type is no longer the fittest virus; their interference with the virus' ability to replicate is such that the virus population will shrink exponentially. Various mutations exist that, if present, reduce the ability of the antiviral drugs to interfere with HIV replication; if they interfere to the extent that the mutant virus population is able to grow overall, the mutation can provide clinically significant resistance.

### Genetic Distance and Fitness Cost

The likelihood of a particular resistance mutation emerging is influenced by two major factors. The first is the relative fitness of the mutation under the current treatment. This may be calculated by considering the relative effectiveness of the mutation at negating the effect of the drug and the relative fitness cost of the mutation. Fitness cost, in this sense, means the decrease in the viruses' ability to effectively replicate in the absence of treatment as a result of the mutation.

The second factor influencing likelihood of emergence is the genetic distance of the resistance mutation from the existing virus pool. This is the number of point mutations necessary to generate the resistance mutation. If the HIV genome is considered to reside in a sequence space, the genetic distance is equivalent to the Hamming distance. Because mutation is a random process, mutations with a high genetic distance are very unlikely to emerge.

#### Example Strains from the Stanford database

Extensive data on resistance mutations to the antivirals listed above has been collected online at the Stanford HIV database [16]. An example from the database can illustrate the genetic distance calculations referenced above. Consider a patient who developed viral resistance to an initial therapy consisting of the NRTIs abacavir and lamivudine and the NNRTI nevirapine (this is a standard first-line therapy). According to the database, one set of mutations yielding significant resistance to these three drugs is (74V,103N,184V), that is, a substitution of valine for leucine in position 74 of the viral reverse transcriptase protein, a substitution of asparagine for lysine in position 103, and a substitution of valine for methionine in position 184. Together, these mutations require at least three point substitutions from the wild-type genome (the number may be higher, as multiple sequences may code for the same amino acid), giving us a genetic distance of 3.

Since the (103N) mutation gives broad class resistance against all NNRTIs, any follow-up therapy will not use NNRTIs. Neither (74V) nor (184V) exhibit strong cross-resistance patterns with any other NRTIs, so a possible follow-up therapy would be the two NRTIs tenofovir and zidovudine together with the PI nelfinavir. Clinically significant resistance to these three drugs could be conferred by the set of mutations (41L,210W,215Y) on the viral RT protein and the mutation (30N) on the viral protease protein. This set of mutations has a genetic distance of 4 from the wild-type, but a genetic distance of 6 from the mutant virus generated in the first round of treatment. This is because the inclusion of either the (74V) or the (184V) mutation increases the susceptibility of the virus to both tenofovir and zidovudine. Consequently, any resistant virus arising to the second treatment will probably arise from the wild-type viral pool, and will probably not carry the (74V), (103N), or (184V) mutations.

Therefore, after developing resistance sequentially to these two treatment regimens, the patient will be carrying three viral strains; the wild-type, one mutant carrying the (74V,103N,184V) RT mutations and another mutant carrying the (41L,210W,215Y) RT mutations and the (30N) Protease mutation,. A resistance mutation to a third antiviral schedule consisting of a permutation of the first two regimens, say abacavir, zidovudine, and nevirapine, would have to arise from one of these parent viruses. The common resistance mutation to this combination with the closest genetic distance to the wild-type carries the mutations (70R, 103N, 184V, 215F) would have a genetic distance of 5. This is also the variant with the shortest genetic distance to the first mutant, with a genetic distance of 4 (the inclusion of the 74V mutation eliminates the resistance to zidovudine, so it must be undone to confer resistance). The variant with the shortest genetic distance to the second mutant would carry the RT mutations (41L,103N,184V,210W,215Y), and would have a genetic distance of either 2 or 3, depending on whether the protease-resistance mutation renders it inviable.

#### Genetic Distance Uncertainty

The computation of genetic distance between HIV strains is relatively straightforward, but there are a few ways in which the genetic distance can be over- or under-estimated. The first is the non-uniqueness of the genetic code; multiple genomic sequences can code for the same amino acid. The calculations carried out in the previous section assumed the parent genome contained the sequence with the shortest genetic distance to the mutant; this may lead to an underestimation of genetic distance.

When using genetic distance to estimate mutational barriers, as described in the next section, the existence of viable transitional forms can result in an overestimation of genetic distance. That is, while the genetic distance to the identified resistant strain may be high, an unidentified partially resistant strain with a lower genetic distance may provide a “stepping-stone” for the development of the fully resistant strain.

## Analysis

### Computation of Risk as a function of Viral Load

#### Pre-existence

The research of Bonhoeffer and Ribeiro [17], [3] show that emergence of resistant virus strains is most likely caused by preexistent resistant mutants under very general conditions. Bonhoeffer [17] also stated that the preexistence does not mean that there is a stable coexistence of sensitive and resistant virus. The preexistence of resistant mutant is made from mutations between sensitive and resistant mutant. Therefore, in order to quantify the risk of drug-resistance emergence, resistance mutations must be modeled as a stochastic process.

#### Poisson Modeling

In this section, equations are presented that determine the drug-resistance emergence risk, which is the mutation probability from the current virus pool to a resistant mutant for the new regimen. To accomplish this, a Poisson distribution is used to model the mutation process. The probability of pre-existing resistant genotypes depends upon two key variables: the total number of active virions for each type of virus present in an infected host, 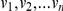, and the point genetic distance from the current virus strains to the emergent resistant mutant 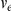: 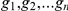. Based on the research of [18], [19],[20], [21], the total viral burden can be estimated. The total viral burden of actively circulating virus can be roughly calculated as the viral titer multiplied by the volume distribution of total body extracellular fluid which is 25-30% of body mass [21]. For example, a 100-kg man (roughly 100-liter volume) with a viral RNA load 10000 copies/ml would have (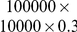) circulating virions approximately.

Therefore, assuming a point mutation rate of 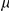, the probability of drug-resistance emergence risk is calculated as follows:



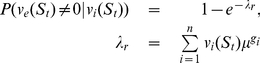
(1)where 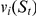 is the viral load of virus subtype 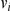 present in the patient at the time of introduction of the naive regimen, 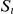.

**Figure 1:**
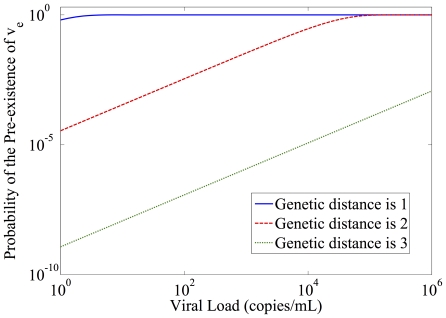
Resistance emergence risk vs. viral load.

Consider the simplest case. Assuming that the current virus pool consists of only one kind of virus strain and the genetic distance from the current virus strain to the resistant mutant is either 1, 2 or 3. Fig. 1 shows the relationship between viral load and the risk of resistant mutant emergence.

As Fig. 1 shows, the resistant mutant may pre-exist when the point genetic distance is 1 or 2, but the pre-existence of resistant mutants is very unlikely if the point genetic distance is 3 or bigger. Consider the case where the current virus are all wild-type and the point genetic distance is 2 between the wild-type virus and a mutant resistant to the naive regimen. If the patient is switched to the naive regimen when the viral load is 30000 copies/ml, the probability that the resistant mutant will preexist is 64.11%. However, if the switch is made when the viral load is 2000 copies/mL, the risk is only 6.86%. Therefore, the task is to create a switch point for the new regimen with the lowest risk.

### Model

To model HIV dynamics, a set of ordinary differential equations is used that includes terms describing the mutations among different virus types. This model depicts the interactions between a wild-type virus population sensitive to all antiviral drug regimens and a resistant mutant virus population only sensitive to treatment with some, if any, antiviral drug combinations. The model is in shown in Equation 2



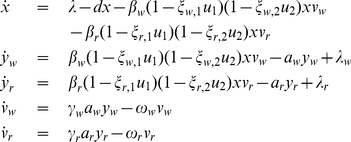
(2)


This model includes states 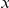, representing CD4+ T cells that are susceptible to infection; 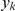, the CD4+ T cells infected by the virus type 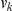, and 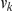, the 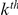 type of virus in the patient's virus pool. 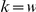 for wild-type virus and 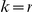 for resistant mutant viruses. The definition of each parameter, and its units, may be found in Table 1.

**Table 1:** State and parameter definitions for Equation 2.

Symbol	Definition	Unit
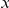	Susceptible CD4+ T cells	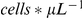
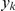	CD4+ T cells infected by the 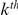 -type virus	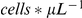
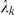	Contribution from the long-lived reservoirs of the 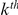 -type virus	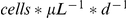
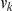	Viral load of the 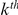 -type virus	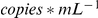
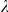	CD4+ T-cell generation rate	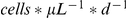
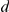	CD4+ T-cell death rate	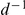
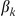	The 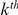 -type virus infection rate	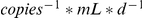
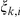	Efficacy of the 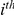 drug regimen on the 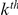 -type virus	
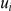	The 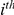 antiviral drug regimen dosing	
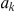	The 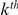 -type infected cell virus-induced death rate	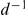
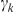	The proliferation rate of the 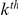 -type virus	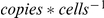
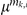	Mutation rate between the 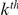 and 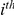 -type virus,	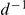
	where the point genetic distance is 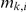 .	
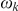	The 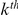 -type virus decay rate	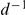

CD4+ T cells are generated from their source at rate 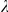 and disappear at rate 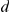. The target cells are infected by the viral strain 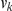 at rate of 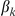 and the therapy suppresses the infection by strain 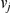 with efficacy 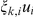, where 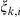 is the maximum effectiveness of antiviral regimen 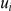 on virus strain 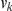.

The infected CD4+ T cells 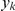 are created first by the infection from target cells 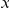 with virus 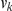 that are generated at a rate 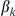. Secondly, there is a contribution due to the activation from long-lived reservoirs at rate 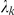. The infected CD4+ T cells die with a rate of 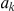.

The virus 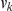 are created from infected CD4+ T cells 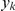. Virus, 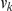, die with a rate of 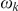. These equations are arbitrarily scalable to any number of viral species.

#### Model identification parameters

To apply the system of equations describing the evolution of viral loads and CD4+T cells to a specific individual, the parameters of the nonlinear differential equations need to be estimated. Using patient data from the AutoVac study [22], a Bayesian estimation technique (specifically, the MCMC, Monte Carlo Markov Chain, method) is used to identify the parameters for this model. In the AutoVac study, HIV patients had viral load measurements taken at a 1-day interval during a series of short treatment interruptions. The data available for estimation is limited to a relatively few values of viral loads after an interruption of medication and reintroduction. In this work, the approach of Huang [23] is used in applying a Bayesian nonlinear mixed-effects model. For the simplified model, there are six parameters to estimate: 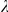, the generation rate of the target cells, 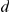, the death rate of the target cells, 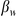, the infection rate for the wild type virus, 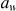, the death rate of the cells infected with the wild type virus, 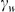, the number of viral particles emitted per infected cell, and 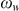, the death rate of viral particles. The generation rates for the wild and resistant virus from long-term reservoirs are assumed to be a small, known constant, and 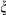 is assumed to be 1. Since the data is so limited, the parameters for the resistant virus are not identified from the data. Instead, they are assumed for the purposes of this paper to be proportional to the parameters for wild-type according to the ratio of viral fitness, with a nominal ratio of 0.5. In practice, this ratio could be estimated from in vitro fitness data available for most common mutation patterns.

**Figure 2:**
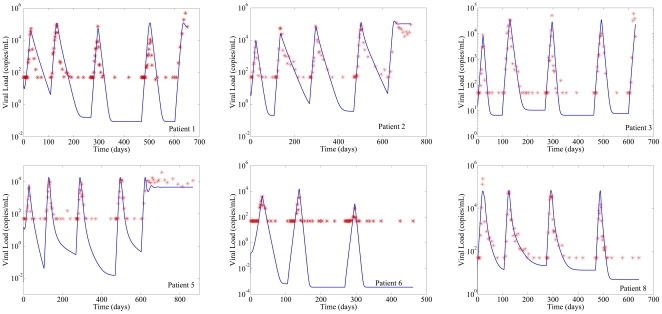
Model fitting for identified patients. Red star: experimental data (detection limit: 50 copies/mL); solid line: estimate. Unless otherwise stated, examples in this paper use parameter values adapted from Patient 1: 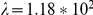, 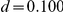, 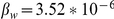, 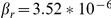, 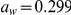, 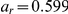, 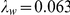, 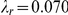, 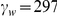, 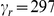, 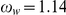, 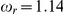.

Parameter estimates were generated for each of 12 patients. For each patient, treatment was interrupted and after a period of time, the treatment was restarted. This cycle of interrupting and reinstating the treatment is repeated 3 or 4 times. The MCMC procedure produced 200,000 possible combinations of parameters that are consistent with the patients' data. From this result, the marginal probability densities for of the six parameters can be established. Among the 12 patients, 3 of them have no detectable virus after an interruption and another 3 appear to be subject to multiple large exogenous disturbances (likely viral blips) which are not accounted for in our model, and result in poor fits. The results of the other 6 patients by using this identification method are shown in Fig. 2.

### Multiple previously failed therapies

In the case of patients who have failed one or more drug regimens previously, the need to preserve the remaining regimens becomes all the more important. Interestingly, the previously failed regimens provide additional control inputs which can be used to reduce the risk of failure for the new regimen at a lower systemic cost than is possible when only one failing regimen is available to use.

#### Regimen Cycling

The multiple failed regimens allow two options for achieving a transient reduction in viral load. Cycling through the previously failed regimens before returning to the currently failing regimen is one such option. Consider a patient with viral dynamics described by Equation 2 who has developed virus strains 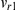 and 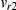 resistant to two previous treatment regimens 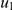 and 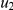. If the viral strains resistant to those regimens are susceptible to the current regimen, they will have decayed to very low levels, and will take some time to re-emerge. Assuming no cross resistance, the currently dominant viral strain is likely susceptible to the original drug regimen 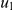 to which the strain 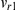 developed resistance. Strictly speaking, so long as 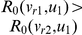 and 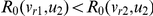, where 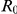 is the basic reproductive ratio defined as



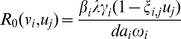
(3)then a transient viral minimum significantly lower than the steady-state level of viral load may be achieved by this method [24].

**Figure 3:**
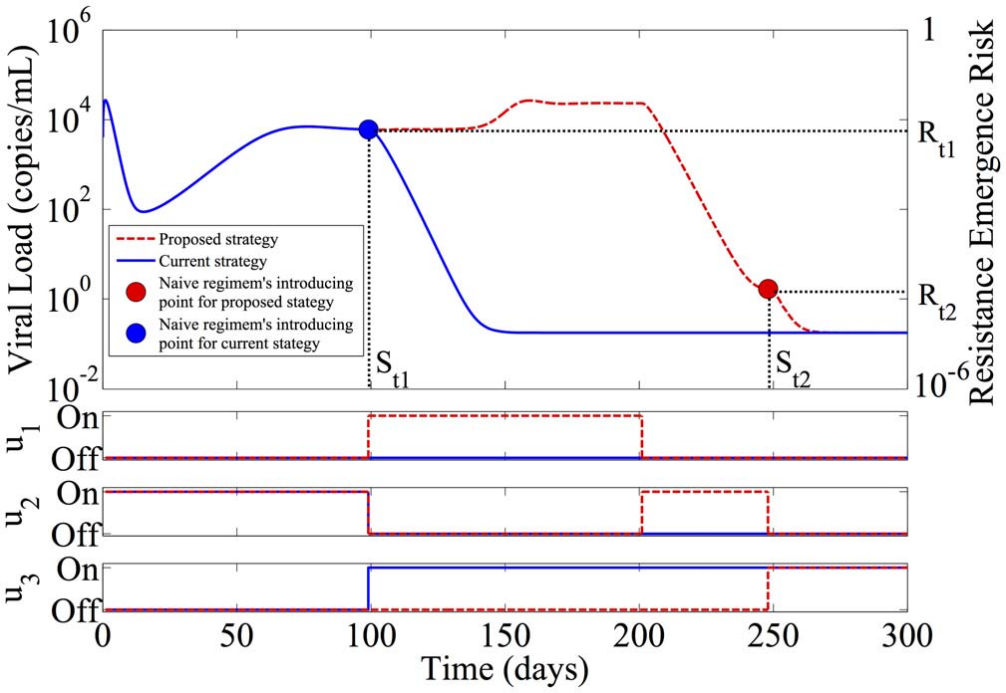
Multiple Previous Failures with Cycling.

Our approach can be formulated as an optimal control problem in two steps. In the first step, the allowable patterns of treatment cycling where either 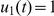 or 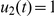 at any time 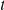 are searched to find a treatment pattern that minimizes the cost function



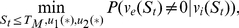
(4)where 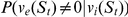 is the cost function defined by Equation 1 and 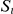 is the time to introduce a naive regimen. In Fig. 2, 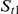 and 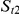 represent the time to introduce a naive regimen in current treatment strategy and our proposed treatment strategy respectively. If the genetic distances between the closest strain resistant to regimen 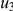 and 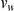,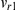, and 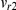, respectively are all equal, this optimization returns the treatment cycling schedule with the largest decrease in total viral load prior to introducing the naive regimen, as seen in Fig. 3. If a naive regimen was not introduced at 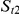, the viral load would rebound shown as red-dash line in Fig. 3. The treatment schedule may be fixed, or may change as the optimization horizon 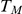 increases, based on the actual values of the parameters in Equation 4 [25]. Since treatment switching on intervals faster than 1 day is not practical, the controls space is discretized at 1 day intervals. This yields an optimization problem with a finite search space, which can be solved in real time using exhaustive search techniques. The second step involves robustly estimating the time at which the minimum in the risk is achieved, and switching to the naive regimen at this point. This study is a subject for future research.

The achievable minimum according to this method will be limited by the initial load levels of the various resistant viruses 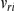. These in turn are determined by the length of time they have been under suppressive therapy, and their relative prevalence in the viral reservoirs. Using a cycling approach to achieve a risk minimum requires tolerating a relatively high viral load for a short period of time, which may contribute to disease progression or increased resistance.

#### Permuted Regimen Introduction

A better option for inducing a transient minimum in the case of multiple previously failed regimens is to introduce a permuted antiviral regimen. It is very likely that that no strain exists which is resistant to permutations of the previously failed therapies. While an antiviral regimen consisting of the permuted components of previous regimens will not provide sufficient mutational barrier to be a viable long-term option, they will allow a dramatic transient reduction in the total viral load, and the corresponding risk of pre-existent resistance.

In the mathematical formulation, the same cost function as Equation 4 is applied. However, the simplified model is not applicable for this case, because 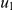 and 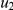 are efficacies of drug cocktails. In this case, 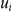 is the efficacy of an individual drug. Therefore, Equation 2 is modified as follows:



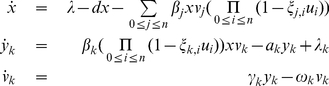
(5)


In this model, there are multiple virus strains, 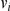, where 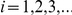, corresponding to wild-type and all existing resistant-type viruses. The variable 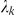 represents the contribution from long-live reservoir, and 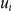 is the efficacy of the 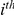 individual drug. The optimization is performed to achieve the minimum cost function defined by Equation 4 by exhaustively searching the possible schedules for each individual drug.

When utilizing permuted regimens, the optimal switching strategy involves switching from the failing regimen directly to a permutation of previously failed regimens prior to introduction of the naive regimen. Every previously dominant strain will be susceptible to this permuted regimen, so this will result initially in exponential decline in the total viral load. However, the reduced genetic distance inherent in using components of previously failed regimens means that resistance to the permuted regimen is likely to emerge. The expected achievable risk reduction can be estimated by assuming that the strain resistant to the permuted regimen pre-exists, with initial conditions related to measures of genetic distance.

**Figure 4:**
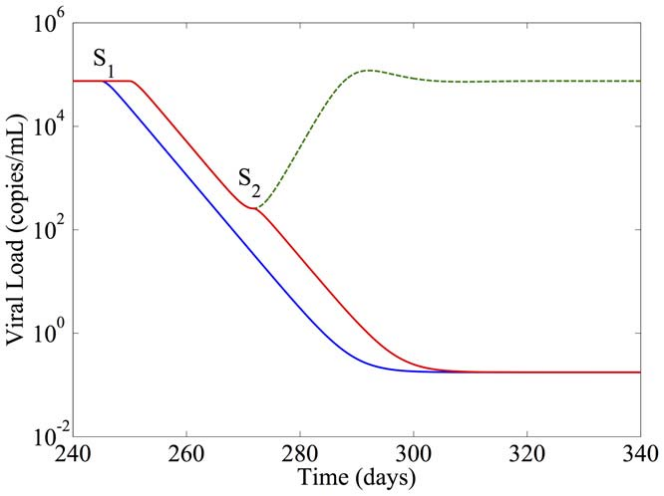
Multiple Previous Failures with Permuted Regimens. Blue line  =  Standard Treatment. Red Line  =  Permuted Regimen Treatment. Green Line  =  Viral Load Rebound.

Assuming that the two previously dominant resistant strains 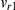 and 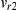 are resistant to drug combinations a+b+c and A+B+C, respectively, then virus resistant to a permuted drug combination such as A+b+c will pre-exist with expected initial viral load



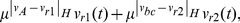
(6)where 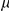 is the pointwise mutation rate for HIV and 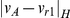 is the number of point mutations in virus variant 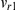 necessary to generate a virus 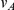 with resistance to drug A and 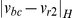 is the number of point mutations in virus variant 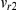 necessary to generate a virus 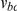 with resistance to drugs b and c, respectively. Note that these are also the Hamming distances applied to the genetic sequences of the respective viruses. Fig. 4 shows the case where only one point mutation separates the dominant resistant strain from a strain resistant to the permuted regimen. Standard treatment introduces a naive regimen at switch point 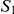. By introducing a permuted regimen at 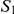, it is possible to achieve a greater than 2 order-of-magnitude reduction in viral load before introducing the naive regimen at 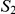. The permuted regimen provides insufficient mutational barrier to prevent resistance, so if a switch is not made, the viral load will rebound. The reduction in resistance emergence risk achieved by this intervention depends on the genetic distance of the dominant strain at the switch time to the closest strain with resistance to the naive regimen. Table 2 illustrates the achievable reduction in risk. It is clear that while a genetic distance of 1 rules out any successful intervention, a genetic distance of 2 or three allow a greater than 2 order-of-magnitude reduction in the risk. Especially in the case of a genetic distance of 2, this is a dramatic change in expected outcome.

**Table 2:** Probability of resistant strain pre-existence at each switch point vs. genetic distance.

Genetic Distance	1	2	3
Switch Point 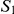	1	0.93	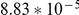
Switch Point 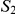	1	0.0089	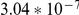

Although current guidelines [26] suggest changing drugs when the virus load exceeds some threshold values (e.g., 1000-5000 copies/mL), finding the exact time when the viral load reaches the threshold is unlikely, because a patient during therapy is only tested for viral load every 3 or 4 months. The equilibrium value of the viral load can therefore be used as the comparison benchmark. It is worth noting that the genetic distance of 1 between the previous viral strains and a strain resistant to the permuted regimen, as used in this example, represents a worst-case scenario (if the genetic distance is 0, this method cannot be used). The example from the Stanford database in the introduction illustrates a real-world case where this distance could be 2 or higher, yielding even greater reductions in viral load and the corresponding risk of pre-existent resistance.

#### Frequent sampling for minimum finding

Both of these methods use optimization to find schedules that create a transient minimum in the total viral load. Successful implementation of these methods require accurately finding the time when this minimum occurs and switching to a naive regimen at that point. The exact time point of the achieved minimum may vary considerably from its calculated point due to parameter variation and the stochastic uncertainty in calculating initial values of emerging resistant virus [27], [28]. Also, a feedback-free application of the schedule would be disastrous if unanticipated resistance to the permuted regimen is present, as this would result in the patient having uncontrolled virus replication for the duration of the schedule. The simplest method to avoid this is sample the viral load frequently following introduction of the permuted regimen, and switch to the naive regimen either when viral load reduction ceases or when a desired reduction in viral load is achieved.

### One Previously failed therapy

The case where a patient has a single previously failed regimen had been the focus of our previous studies [29]. The only strategy that yields effective reduction of future mutation risk in this case involves total treatment interruptions.

#### Treatment interruptions and optimal scheduling

Our objective is to find a drug-switching schedule that yields the minimum risk, which is calculated as shown in the method in the Analysis section. For patients with only a single previously failed regimen, this can be achieved only through the use of interrupted schedules of treatment. The concept is identical to that driving the regimen cycling approach described in the previous section, except that periods of no treatment are allowed. If the resistant virus generated during the previous therapy has associated fitness cost with respect to the wild-type virus, then these periods of no treatment allow the wild-type virus to re-establish dominance. Re-introduction of the failing regimen will then result in a transient decrease in total viral load before the resistant strain re-establishes dominance.

As in the previous section, this is an optimal control problem in two steps. The optimal schedules consist of an interruption of length 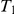, to sensitize the virus, followed by the re-introduction of the failing regimen, resulting in transient suppression of the sensitized viral load. Before viral load rebound occurs, the naive antiviral regimen is introduced at time 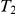, resulting in a greatly reduced risk of subsequent virological failure. 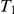 is again calculated to minimize the cost function of Equation 4, and 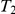 is the switching time that achieves this minimum.

**Figure 5:**
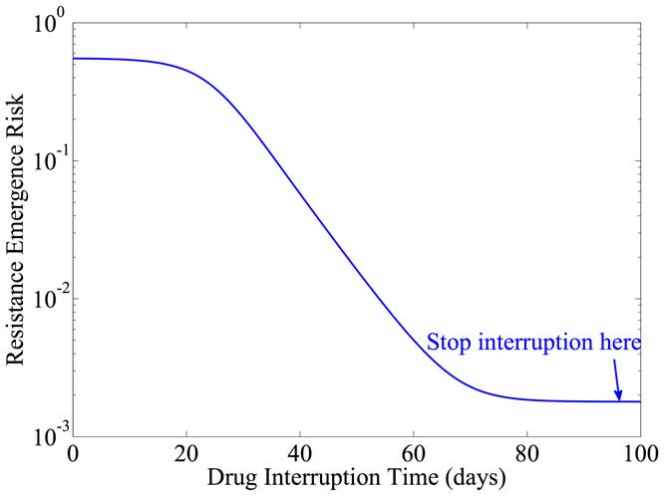
Achievable risk reduction as a function of interruption length.

**Figure 6:**
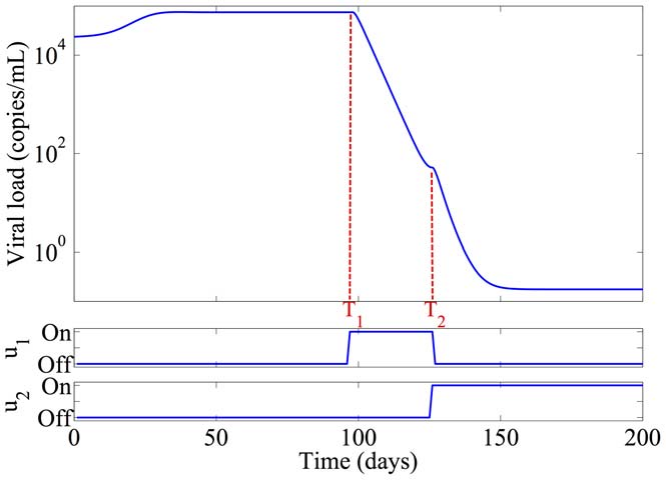
Optimal switching schedule. Treatment is interrupted at time 0,and reintroduced at time 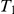. At time 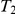, naive treatment is introduced, yielding an 2 order-of-magnitude reduction in risk.

An example is used to illustrate how our algorithm works. The simplified model of Equation 2 is employed with the parameters which are identified for Patient 1. Assume that the point genetic distances 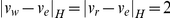. The optimal time for introduction of the naive regimen is fixed by the initial conditions at time 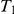. The optimal interruption length 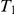 changes as a function of parameters; there may be a true optimum, or the optimal time may be infinite, as shown in Fig. 5 [25]. Where there is no minimum, the knee in the curve, where increased interruption time yields only marginal benefit, dictates the switching time. Fig. 6 shows the associated optimal switching schedule, with viral load as a function of time. In this case, the risk of resistance emergence is reduced from almost 55.4% to 0.18% by applying our algorithm.

#### Evolutionary Risks

Treatment interruption regimens have been associated with a high rate of resistance emergence [30], and are consequently avoided in standard HIV therapy [26]. Careful analysis of their use in this context is needed to avoid the possibility of encouraging development of multi-drug resistant viral strains.

## Discussion

We have presented methods to reduce the risk of drug resistance emergence in HIV by manipulating the viral load prior to introduction of a naive antiviral regimen. These methods rely on creating a transient reduction in total viral load prior to introduction of a naive regimen. If the patient has failed multiple previous regimens, this may be accomplished either through regimen cycling or the use of a permuted antiviral regimen. If the patient has failed only a single previous regimen, this may be accomplished through the use of interrupted therapy. The optimal switching regimens are computed using simple model-based open-loop optimal control algorithms. In all cases, the models predict achievable order-of-magnitude reduction in the risk of resistance to the naive regimen pre-existing its introduction.

The method proposed in this paper uses predictive models of HIV evolution under conditions of dynamic treatment to find treatment schedules that minimize the probability of certain resistant strains emerging. The application of dynamical systems and control to evolutionary systems will likely find broader application, as problems of drug resistance continue to increase in other areas of medicine.

While risk reduction should be achievable through any of these three methods, regimen cycling and interrupted therapy carry an increased risk of disease progression and/or further development of resistant virus. For these reasons, initial clinical investigations will focus primarily on the method of permuted regimens. Nevertheless, the other two methods may be useful in certain circumstances.

The problem of multi-drug resistance in HIV is extremely widespread, and methods that preserve remaining suppressive antiviral regimens have the potential to significant decrease morbidity and mortality in the HIV-positive population. The necessary next steps for implementing this method are outlined below:

### Implementation Issues and Future Works

The methods introduced in this paper have the potential to significantly reduce the incidence of pre-existence related treatment failure. The methods involving permuted regimens could be applied to select groups of patients without significant further work. These patients would have to be in a closely monitored clinical situation, have a history of consistent viral genotyping showing strain patterns amenable to this method, and would have to be available for frequent viral load sampling. For these methods to be more broadly applied, several issues involving measurement, viral load history, and sampling frequency will need to be addressed.

#### Unmodeled Phenomenon

The model of Equation 2 represents a highly reduced form of the HIV dynamics, and neglects many known factors in HIV infection. Perhaps chief among these is the immune response to HIV, which can change the infection rate 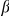 and the death rate of infected cells 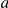 significantly. There is some previous work suggesting that the immune response can change dynamically with respect to changes in viral load, and that the consequences of this can be significant, potentially leading to immunological control of the virus under some circumstances [31]–[33]. However, multiple previous experiments summarized in [34] show that, once the chronic infection stage is reached, the immune system is permanently damaged and no longer displays such dramatic dynamic response to changing viral loads. The excellent fits to the simplified model over several successive treatment interruptions shown in Fig. 2 argues strongly for the sufficiency of the model of Equation 2 to predict the dynamics of HIV during therapy changes. Furthermore, the algorithm proposed in this paper does not actually depend on the exact form of the model, but uses a closed-loop sampling method to find the viral load minimum, and is therefore robust to unmodeled phenomenon such as a changing immune response.

#### Measurement issues

The methods described in this paper rely heavily on the frequent and accurate measurement of the HIV viral load, both for risk computation and model identification. Viral load measurements are complicated by the existence of a detection limit of 50 copies/mL. Targeting viral load reduction below the limit of detection is therefore problematic. However, reduction of viral load to this limit of detection is sufficient in most cases to achieve a significant order-of-magnitude reduction in risk.

A second issue related to measurement is the proportion of the measured viral load which is noninfectious. Cells with defective integrated viral genomes may produce replication-incompetent virus particles, and a proportion of the particles produced by all infected cells will also be replication-incompetent. The most direct measurement of this phenomenon estimates that between 5–13% of the total free virus particles are capable of successfully completing the infection process (a much lower percentage actually complete the process, due to high inefficiencies at each intermediate step of replication) [35]. If a model of HIV dynamics is identified against plasma HIV RNA quantifications, which do not differentiate between infectious and noninfectious particles, then the estimate of the infection rate parameter 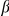 will implicitly be multiplied by the percentage of plasma virions which are infectious. This will not be a problem so long as all measurements are consistent between the model identification and prediction; estimated values of the number of infected cells will not be affected. The method of action of protease inhibitors results in an increase in the percentage of viruses which are noninfectious; this reduced fraction will be implicitly captured in the estimated value of the drug efficacy parameter 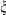 associated with the PI-containing regimen. The proportion of measured virus that is non-infectious will not affect the risk reduction algorithm, as the algorithm attempts to minimize the measured virus prior to switching, which is always proportional to the infectious virus. This phenomenon will slightly alter the calculated risk associated with a given measured viral load, but as the risk only depends logarithmically on the viral load, the change due to this relatively small proportional difference will be negligible.

A third issue, related to the second, is the fact that plasma viral concentrations are not the same as the virus concentrations in the lymphoid tissues, where the majority of the HIV virus resides and the where the majority of virus dynamics occur. HIV virus penetrates into many different tissues in the body, and there is evidence that these tissues are sufficiently compartmentalized to allow for divergent evolution of the virus in different compartments [18]–[20].The conversion factor developed in [21] and used in Equation 1 is a good first order approximation of the relationship between plasma virus level and total viral burden. A recent study in SHIV infected Rhesus Macaques has shown excellent proportional correlation between viral concentrations in plasma and various other tissue types both under treated and untreated conditions [36], indicating that the plasma virus load is a good surrogate measurement of total viral burden, even under conditions of dynamic therapy. The exact ratio between plasma viral load and total viral burden will change from patient to patient, but this will not affect the proposed algorithm, though it will slightly alter the calculated risk associated with a measured viral load, as discussed above.

#### Choosing drug permutations

The most promising method presented in this paper is the introduction of permuted antiviral regimens prior to the introduction of a naive regimen. In order to safely choose these permuted regimens, it is necessary to know which resistant viruses are present in the patient's viral reservoirs. Consistent viral genotyping following every treatment failure would provide this information; unfortunately, this is rare. It may be possible to estimate the likely distribution of resistant viruses in a patient based on a history of antiviral use and failure, using genetic distance and fitness information from the HIV database.

#### Finding Minima

All the methods presented in this paper induce a transient crash in the viral load, and depend on being able to switch therapies at or near the minimum of this crash, prior to rebound. In this paper, the method suggested for this is consistent, frequent sampling of the viral load during the transient period. While this should work, it is expensive both in economic terms and in terms of patient burden. Optimal minimal sampling methods to find the viral load minima with the fewest possible measurements should solve this issue; this research is ongoing [37]–[39].

## References

[1] Gray C, Lawrence J, Ranheim E, Vierra M, Zupancic M (2000). Highly active antiretroviral therapy results in HIV type 1 suppression in lymph nodes, increased pools of naive T cells, decreased pools of activated T cells, and diminished frequencies of peripheral activated HIV type 1-specific CD8+ T cells.. AIDS Res Hum Retroviruses.

[2] Ho DD, Neumann AU, Perelson AS, Chen W, Leonard JM (1995). Rapid turnover of plasma virions and CD4 lymphocytes in HIV-1 infection.. Nature.

[3] Ribeiro RM, Bonhoeffer S (2000). Production of resistant HIV mutants during antiretroviral therapy.. Proc Natl Acad Sci USA.

[4] Ribeiro R, Mohri H, Ho D, Perelson A (2002). In vivo dynamics of T cell activation, proliferation, and death in HIV-1 infection: why are CD4+ but not CD8+ T cells depleted?. Proc Natl Acad Sci U S A.

[5] Hammer SM, Saag MS, Schechter M, Montaner JSG, Schooley RT (2006). Treatment for adult HIV infection: 2006 recommendations of the International AIDS Society-USA panel.. JAMA.

[6] Deeks S, Wrin T, Liegler T, Hoh R, Hayden M (2001). Virologic and immunologic consequences of discontinuing combination antiretroviral-drug therapy in HIV-infected patients with detectable viremia.. New Engl J Med.

[7] Deeks SG, Grant RM, Wrin T, Paxinos EE, Liegler T (2003). Persistence of drug-resistant HIV-1 after a structured treatment interruption and its impact on treatment response.. AIDS.

[8] Deeks SG, Hoh R, Neilands TB, Liegler T, Aweeka F (2005). Interruption of treatment with individual therapeutic drug classes in adults with multidrug-resistant HIV-1 infection.. J Infect Dis.

[9] Ghosn J, Wirden M, Ktorza N, Peytavin G, Aït-Mohand H (2005). No benefit of a structured treatment interruption based on genotypic resistance in heavily pretreated HIV-infected patients.. AIDS.

[10] Benson CA, Vaida F, Havlir DV, Downey GF, Lederman MM (2006). A randomized trial of treatment interruption before optimized antiretroviral therapy for persons with drug-resistant HIV: 48-week virologic results of ACTG A5086.. J Infect Dis.

[11] Katlama C, Dominguez S, Gourlain K, Duvivier C, Delaugerre C (2004). Benefit of treatment interruption in HIV-infected patients with multiple therapeutic failures: a randomized controlled trial (ANRS 097).. AIDS.

[12] Lawrence J, Mayers DL, Hullsiek KH, Collins G, Abrams DI (2003). Structured treatment interruption in patients with multidrug-resistant human immunodeficiency virus.. N Engl J Med.

[13] Bonjoch A, Buzon MJ, Llibre JM, Negredo E, Puig J (2008). Transient treatment exclusively containing nucleoside analogue reverse transcriptase inhibitors in highly antiretroviral-experienced patients preserves viral benefit when a fully active therapy was initiated.. HIV clinical trials.

[14] Martinez-Picado J, Negredo E, Ruiz L, Shintani A, Fumaz CR (2003). Alternation of antiretroviral drug regimens for HIV infection. A randomized, controlled trial.. Ann Intern Med.

[15] Mansky LM (1996). Forward mutation rate of human immunodeficiency virus type 1 in a T lymphoid cell line.. AIDS Res Hum Retroviruses.

[16] Shafer RW (2006). Rationale and uses of a public HIV drug-resistance database.. J Infect Dis.

[17] Bonhoeffer S, Nowak MA (1997). Pre-existence and emergence of drug resistance in HIV-1 infection.. Proc Biol Sci.

[18] Korber BT, Kunstman KJ, Patterson BK, Furtado M, McEvilly MM (1994). Genetic differences between blood- and brain-derived viral sequences from human immunodeficiency virus type 1-infected patients: evidence of conserved elements in the V3 region of the envelope protein of brain-derived sequences.. J Virol.

[19] Zhu T, Wang N, Carr A, Nam DS, Moor-Jankowski R (1996). Genetic characterization of human immunode_ciency virus type 1 in blood and genital secretions: evidence for viral compartmentalization and selection during sexual transmission.. J Virol.

[20] Wong JK, Ignacio CC, Torriani F, Havlir D, Fitch NJ (1997). In vivo compartmentalization of human immunode_ciency virus: evidence from the examination of pol sequences from autopsy tissues.. J Virol.

[21] Colgrove R, Japour A (1999). A combinatorial ledge: reverse transcriptase fidelity, total body viral burden, and the implications of multiple-drug HIV therapy for the evolution of antiviral resistance.. Antiviral Res.

[22] Ruiz L, Carcelain G, Martínez-Picado J, Frost S, Marfil S (2001). HIV dynamics and T-cell immunity after three structured treatment interruptions in chronic HIV-1 infection.. AIDS.

[23] Huang Y, Liu D, Wu H (2006). Hierarchical Bayesian methods for estimation of parameters in a longitudinal HIV dynamic system.. Biometrics.

[24] Zurakowski R, Wodarz D (2007). Treatment interruptions to decrease risk of resistance emerging during therapy switching in HIV treatment.. Proc. of the 46th IEEE Conference on Decision and Control.

[25] Luo R, Zurakowski R (2008). A new strategy to decrease risk of resistance emerging during therapy switching in HIV treatment.. Proc. of the American Control Conference.

[26] Hammer SM, Eron JJ, Reiss P, Schooley RT, Thompson MA (2008). Antiretroviral treatment of adult HIV infection: 2008 recommendations of the International AIDS Society-USA panel.. JAMA.

[27] Luo R, Piovoso M, Zurakowski R (2010). Modeling-error robustness of a viral-load preconditioning strategy for HIV treatment switching.. Proc. of the American Control Conference.

[28] Luo R, Cannon L, Hernandez J, Piovoso MJ, Zurakowski R (2011). Controlling the evolution of resistance.. Journal of Process Control.

[29] Luo R, Piovoso M, Zurakowski R (2009). A generalized multi-strain model of HIV evolution with implications for drug-resistance management.. Proc. of the American Control Conference.

[30] Ananworanich J, Nuesch R, Braz ML, Chetchotisakd P, Vibhagool A (2003). Failures of 1 week on, 1 week off antiretroviral therapies in a randomized trial.. AIDS.

[31] Wodarz D (2001). Helper-dependent vs. helper-independent CTL responses in HIV infection: implications for drug therapy and resistance.. J Theor Biol.

[32] Lifson J, Rossio J, Arnaout R, Li L, Parks T (2000). Containment of simian immunodeficiency virus infection: cellular immune responses and protection from rechallenge following transient postinoculation antiretroviral treatment.. J Virol.

[33] Lifson JD, Rossio JL, Piatak M, Parks T, Li L (2001). Role of CD8(+) lymphocytes in control of simian immunodeficiency virus infection and resistance to rechallenge after transient early antiretroviral treatment.. J Virol.

[34] Zurakowski R (2011). Nonlinear observer output-feedback MPC treatment scheduling for HIV.. Biomed Eng Online.

[35] Thomas JA, Ott DE, Gorelick RJ (2007). Efficiency of human immunodeficiency virus type 1 postentry infection processes: evidence against disproportionate numbers of defective virions.. J Virol.

[36] North TW, Higgins J, Deere JD, Hayes TL, Villalobos A (2010). Viral sanctuaries during highly active antiretroviral therapy in a nonhuman primate model for AIDS.. J Virol.

[37] Rosero-Garcia E, Zurakowski R (2010). Closed-loop minimal sampling method for determining viral-load minima during switching.. Proc. of the American Control Conference.

[38] Cardozo EF, Zurakowski R (2011). Measurement error robustness of a closed-loop minimal sampling method for HIV therapy switching.. Proc. of the IEEE Eng. Medicine and Biology Conference.

[39] Zurakowski R, Churgin M, Perez C, Rodriguez M (2011). Approximate-model closed-loop minimal sampling method for HIV viral-load minima detection.. Proc. of the American Control Conference.

